# Dr. Henry J. Bigelow, and the Discovery of Anæsthesia

**Published:** 1891-04

**Authors:** 


### Dr. Henry J. Bigelow and the Discovery of Anæsthesia.

In a speech delivered at a memorial meeting of the Boston Society for Medical Improvement, on the occasion of the death of Dr. Henry J. Bigelow, Dr. O. W. Holmes said:

Dr. Bigelow sometimes paid me the compliment of asking my opinion of, and my criticism upon, an essay or a lecture he was about to read or publish. On an evening of December, 1846, he called upon me with a paper which he proposed reading the next evening at the regular meeting of the American Academy of Arts and Sciences. He began by telling me that a great discovery had just been made and practically demonstrated in the operating theatre of the Massachusetts General Hospital. He proceeded to read the paper, which was the first formal presentation to the world of the successful use of artificially produced anæsthesia in a capital operation. He had the sagacity to see the far-reaching prospects of the new discovery, the courage as well as the shrewdness to support the claims of the adventurous dentist's startling at first almost incredible, announcement. Every possible effort was made to dislodge the infant anæsthesia from its cradle in the Massachusetts Hospital, but there remains the fact that all over the wide world patients were shrieking under the surgeon's knife and saw—operator and victim alike ignorant of the relief in store for them, at the very time when Dr. Bigelow was unfolding in my library the first paper ever written on the subject, and saying to me as he did so, that within a fortnight the news of the discovery would be all over Europe. From the first, Dr. Bigelow was the steady, unflinching advocate of ether as the safest of the anæsthetics, and his views, though not universally accepted, have had a very wide and lasting influence.—*Northwestern Lancet.*

